# Magnetic Zr-Based Metal-Organic Frameworks: A Highly Efficient Au (III) Trapper for Gold Recycling

**DOI:** 10.3390/ma15196531

**Published:** 2022-09-21

**Authors:** Ziyong Chang, Xiaosha Gong, Liang Zeng, Junlian Wang, Yangge Zhu

**Affiliations:** 1 Civil and Resource Engineering School, University of Science and Technology Beijing, Beijing 100083, China; 2 BGRIMM Technology Group, State Key Laboratory of Mineral Processing, Beijing 100160, China

**Keywords:** magnetic, metal-organic frameworks, adsorption, Au (III), molecular dynamics simulation

## Abstract

In this work, the magnetic Zr-based MOF composites with excellent retrievability were prepared using Fe_3_O_4_@SiO_2_ as the core and UiO–66–NH_2_ as the shell. Fe_3_O_4_@SiO_2_ core could introduce mesopores and result in capillary condensation in MOF composites, which aggravated with the dosage of Fe_3_O_4_@SiO_2_. The as-synthesized MOF composites could be rapidly retrieved from aqueous solution via magnetic separation in 10 seconds. pH imposed an important effect on Au (III) adsorption by governing the ion exchange and electrostatic interaction between Au (III) anions and adsorbents, and the optimal adsorption happened at pH 7. The adsorption process fitted well with the pseudo-second order kinetics model and Langmuir adsorption model. The maximum adsorption capacity of Au (III) by FSUN–10 and FSUN–50 at 298 K were determined to be 611.18 mg∙g^−1^ and 463.85 mg∙g^−1^, respectively. Additionally, Au (III) uptakes increased with temperature. Beyond experiments, the adsorption mechanisms were thoroughly studied through systematic characterization, molecular dynamics simulation (MDS) and density functional theory (DFT) study. It was verified that Au (III) was adsorbed via coordination to hydroxyl and amino groups and was reduced to Au (I) and Au (0) by amino groups. The diffusion coefficient of Au (III) along UiO–66–NH_2_ was calculated to be 5.8 × 10^−5^ cm^2^∙s^−1^. Moreover, the magnetic Zr-based MOF composites exhibit great industrial value in gold recycling with high adsorption selectivity and good recyclability.

## 1. Introduction

Gold is widely utilized in jewelry, medical, chemical, electronic and electrical industries due to its excellent physical and chemical properties [1]. However, the demand of gold is increasing rapidly due to the sustainable development of industry and improvement of consumption. Nowadays, recycling gold from secondary resources has been the priority choice for countries around the world to cope with the resource shortage [2]. Recovering gold from secondary resources is more effective, much cleaner and less costly compared to mining and metallurgy, which is of great environmental and economic significance [3].

Adsorption is widely used in the preconcentration and recovery of gold from aqueous solution since it has the advantages of being a short process, a clean working environment and easy to operate [4]. Activated carbons (ACs), resin and biomaterials are conventional adsorption materials for gold recycling. ACs can effectively adsorb gold ions through physical and chemical interactions and have a high stability in harsh environments [5]. However, the main limitation is that ACs are difficult to regenerate after use, and the adsorption capacity and selectivity are relatively low. Resin is easier to be regenerated than ACs and has higher adsorption efficiency while the adsorption capacity and selectivity are limited [6]. Biomaterials such as algae, fungi, yeasts and some biopolymers are environment friendly and low cost, and have also been utilized in the adsorption and recovery of gold [7]. However, the disadvantages of biomaterials are limited adsorption capacity and poor stability, thus restricting their large-scale application in practice. Therefore, developing adsorption materials with large capacity, high efficiency and superb selectivity is vital to gold extraction and recycling.

Metal-organic frameworks (MOFs) are emerging as promising candidate adsorbents for gold recycling. MOFs are composed of inorganic metal ions and organic linkers by coordination via self-assembly. MOFs are periodic porous materials with tunable topology and pore diameter, enormous surface area and versatile chemical properties. Therefore, MOFs are widely studied in multiple applications, such as energy storage [8], biomedicine [9,10], wastewater treatment [11,12] and catalysis [13]. In the last decades, MOFs have gained a great number of attention in the capture and adsorption of precious metals. Hu [14] reported that the maximum adsorption capacity of Au (III) on zeolitic imidazolate framework (ZIF-8) was 1192 mg∙g^−1^ at pH 2.5. Tang et al. [15] synthesized AHPP-MOF via premodification to effectively adsorb Pd (II) from aqueous solution with the maximum uptakes being 241.6 mg∙g^−1^ at 298 K. In a previous study, authors found that UiO–66–NH_2_ exhibited exceptional adsorption selectivity and efficiency towards Au (III) [16]. However, the submicron powdery shape of MOFs requires tedious high-speed centrifugation to achieve solid–liquid separation, seriously limiting their application in practice. In addition, though great efforts have been devoted in employing Zr-based MOFs for Au (III) recovery, the underlying adsorption mechanism, especially when reducing groups (i.e., -NH_2_) exist in MOF, is still yet to be revealed.

The retrieval of MOFs from aqueous solution can be facilitated by functionalization with superparamagnetic iron oxide nanoparticles. Typically, the magnetic nanocomposites composed of Fe_3_O_4_ nanoparticles and MOFs can be prepared via layer-by-layer assembly strategy to generate a core-shell structure. For example, Hu et al. [17] synthesized hybrid magnetic MOF-5 by the incorporation of Fe_3_O_4_ nanoparticles, making them the ideal candidate adsorption materials for magnetic enrichment of trace analytes. Moreover, silica layer was usually adopted to stabilize the Fe_3_O_4_ core in harsh aqueous environment, and the -OH groups on silica could facilitate the in-situ growth of MOF shell by chelating with metal ions. Huang et al. [18] prepared magnetic MOFs with nano-sized Fe_3_O_4_@SiO_2_ as core and Zr-based MOFs as shell, which showed high adsorption capacity and fast adsorption kinetics towards heavy metal ions and organic dyes. 

**Scheme 1:**
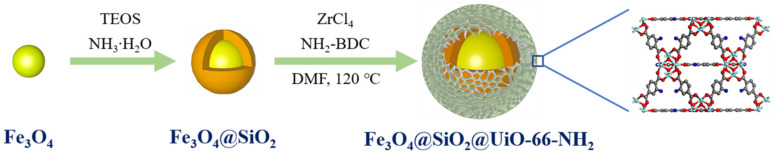
Synthesis of core-shell magnetic MOF composites.

Herein, based on the previous study, we prepared magnetic MOF composites with Fe_3_O_4_@SiO_2_ as the core and UiO–66–NH_2_ as the shell to adsorb Au (III) from aqueous solution (Scheme 1). Effects of magnetic functionalization on the structure of MOF composites and their adsorption performance were investigated thoroughly, and the underpinning adsorption mechanisms were studied through experimental characterization, molecular dynamics simulation (MDS) and density functional theory (DFT) study. 

## 2. Experimental

### 2.1. Synthesis Methods

#### 2.1.1. Fe_3_O_4_ Synthesis

In this work, Fe_3_O_4_ nanoparticles were synthesized based on the modification of a previously reported hydrothermal method [19]. Specifically, 10 mL 0.3 M FeCl_3_∙6H_2_O aqueous solution was added into 20 mL 0.45 M NaHCO_3_ solution and conditioned for 30 min. Then, 10 mL 0.05 M vitamin C (VC) solution was added dropwise into the mixture. The molar ratio of VC to Fe^3+^ was kept as 1:6. Subsequently, the mixture was transferred into a 50 mL steel-lined para-polyphenylene (PPL) autoclave and heated at 180 °C for 8 h. The obtained particles were washed with deionized water and ethanol repeatedly, followed by drying at 80 °C overnight.

#### 2.1.2. Fe_3_O_4_@SiO_2_ Synthesis

The core-shell structured Fe_3_O_4_@SiO_2_ nanoparticles were prepared using a sol–gel method [20]. 230 mg Fe_3_O_4_ nanoparticles were dispersed into a mixture consisting of 80 mL ethanol, 20 mL ultrapure water and 1.5 mL NH_3_∙H_2_O solution (25–28 wt.%) by ultrasonic water bath for 30 min. The pH of this mixture was kept at 8–10. After that, 0.5 mL tetraethyl orthosilicate (TEOS) was introduced into the abovementioned mixture at a speed of 10 μL·min^−1^. The reaction was conducted for 12 h under vigorous mechanical stirring and the obtained particles were collected using a magnet, followed by washing with ethanol and ultrapure water for three times. The products, denoted as Fe_3_O_4_@SiO_2_, were redispersed in N, N-dimethyformamide (DMF) for the subsequent preparation of magnetic functionalized MOFs.

#### 2.1.3. Synthesis of Fe_3_O_4_@SiO_2_@UiO–66–NH_2_ (FSUN) 

FSUN was prepared using a solvothermal method. Briefly, 80 mg ZrCl_4_, 62 mg 2-aminoterephthalic acid (NH_2_-BDC) and 1.5 mL glacial acetic acid were mixed with 50 mL DMF. Fe_3_O_4_@SiO_2_ dispersion (10 mg∙mL^−1^) was added into the abovementioned mixture gradually, followed by the ultrasonic water bath for 30 min. Then the mixture was transferred into a steel lined PPL autoclave and heated at 120 °C for 24 h. The harvested products were collected by magnetic separation and washed with DMF and ethanol repeatedly, followed by drying at 80 °C overnight. To prepare FSUN with different magnetism, 1 mL (10 mg) and 5 mL (50 mg) Fe_3_O_4_@SiO_2_ dispersion were used and the resultant products were denoted as FSUN–10 and FSUN–50, respectively.

### 2.2. Adsorption Test

For each test, 3 mg FSUN particles and 3 mL Au (III) aqueous solution were mixed in a 5 mL EP tube and incubated at 200 rpm. The pH of mixture was regulated to the desired value using 0.1 M HCl or NaOH solution. The adsorbents were retrieved by magnetic separation after adsorption, and the residual Au (III) concentration was determined by ICP-OES. Au (III) uptakes by FSUN were calculated as Equations (1) and (2):

(1) $$q_{e}=\frac{(C_{0}-C_{e})\cdot V}{m}$$

(2) $$q_{t}=\frac{(C_{0}-C_{t})\cdot V}{m}$$

where *q_e_* (mg∙g^−1^) and *q_t_* (mg∙g^−1^) are Au (III) uptakes at equilibrium and time *t* (min), *V* (L) is the volume of aqueous solution for each test, *C*_0_ (mg∙L^−1^) and *C_t_* (mg∙L^−1^) are the initial Au (III) concentration and residual Au (III) concentration at time *t* (min), and *m* (g) is the dosage of FSUN used for each test.

To study the adsorption kinetics, the initial Au (III) concentration was fixed at 400 mg∙L^−1^ with the incubation time ranging from 30 min to 24 h. The adsorption data were fitted by the pseudo-first order and pseudo-second order kinetics model, respectively. The expressions of the above adsorption kinetics models are as Equations (3) and (4):

(3) $$q_{t}=q_{e}(1-\exp(-k_{1}\cdot t))$$

(4) $$q_{t}=\frac{k_{2}\cdot q_{e}^{2}\cdot t}{1+k_{2}\cdot q_{e}\cdot t}$$

where *k*_1_ (min^−1^) and *k*_2_ (g∙mg^−1^∙t^−1^) are pseudo-first order kinetics rate constant and pseudo-second order kinetics rate constant, respectively.

The adsorption isotherms and thermodynamics were investigated by varying the initial Au (III) concentration from 100 mg∙L^−1^ to 1600 mg∙L^−1^, and the temperature ranged from 298 K to 318 K. The adsorption data were fitted by Langmuir and Freundlich models with expressions as Equations (5) and (6):

(5) $$q_{e}=\frac{K_{L}\cdot q_{m}\cdot C_{e}}{1+K_{L}\cdot C_{e}}$$

(6) qe=KF⋅Ce1nF

where *q_m_* (mg∙g^−1^) is the theoretical maximum Au (III) uptakes, *K_L_* (L∙mg^−1^) is the Langmuir adsorption constant, *K_F_* and *n_F_* are the Freundlich adsorption parameters.

### 2.3. Reusability Study

In this work, to study the reusability of FSUN as adsorbents, 30 mg FSUN was incubated with 100 mg∙L^−1^ Au (III) solution initially. After each cycle, the used adsorbents were collected by magnetic separation. Then the metal-loaded adsorbents were eluted using 5 mL 0.02 M acidic thiourea three times (15 mL in total), then washed with 5 mL ultrapure water for three times (15 mL in total). The regenerated adsorbents were dried in vacuum oven at 80 °C overnight and reused for batch adsorption tests.

### 2.4. Computational Simulation

#### 2.4.1. MDS Study

The crystallographic information file (cif) of UiO–66–NH_2_ used for MDS study was obtained from Cambridge Crystallographic Data Centre (CCDC) [21]. The molecular structure of AuCl_4_^−^, H^+^ and H_2_O (Figure S1) were constructed by ChemDraw. The MDS study of AuCl_4_^−^ adsorption by UiO–66–NH_2_ was carried out using the Forcite module installed in Materials Studio. Initially, a 2 × 2 × 2 supercell of UiO–66–NH_2_ with lattice length of 40.97 Å × 40.97 Å × 40.97 Å was built and geo-optimized. The guest molecules including 10 AuCl_4_^−^ molecules, 10 H^+^ and 100 H_2_O molecules were put into a box through Amorphous Cell module. Then UiO–66–NH_2_ and guest molecules were put together to build a sandwich structure (Figure S1e) using the layer tool. The lattice length of this box was 40.97 Å × 40.97 Å × 58.07 Å. Geometry optimization was conducted using smart method and the threshold values for convergence tolerance were 0.001 kcal∙mol^−1^ for the maximum energy, 0.5 kcal∙mol^−1^∙Å^−1^ for the maximum force and 0.015 Å for maximum displacement. The adsorption dynamics was simulated using NVT ensemble and Nose thermostat at 298 K. The dynamics simulation completed within 500 ps at a timestep of 1.00 fs. During the dynamic simulation process, the Cartesian position of UiO–66–NH_2_ was fixed and only guest molecules were allowed to move freely. Universal Forcefield (UFF) was adopted in the whole process of geometry optimization and dynamics simulation.

Additionally, the dynamics behavior of AuCl_4_^−^ adsorption was described and analyzed using mean squared displacement (MSD), which was calculated as Equation (7):

(7) $$MSD(t)=\frac{1}{N}\sum_{i=1}^{N}{\left(|r_{i}(t+t_{0})-r(t_{0})|\right)}^{2}$$

where *r_i_* (t) is the position of atom i at time t and *N* is the amounts of atoms. Thereby, the diffusion coefficient (*D*) of guest molecules along the framework could be calculated using Equation (8) [22]:

(8) $$D=\frac{1}{6}\underset{t\to \infty }{\lim}\frac{dMSD}{dt}$$

#### 2.4.2. DFT Study

The DFT study was conducted using the CASTEP module installed in Materials Studio. The lowest energy was determined by Sorption locator. GGA/PBE functional was adopted for electron calculation and ultrasoft pseudopotential was used to study the interactions between guest molecules and UiO–66–NH_2_. The cutoff energy was set as 380 eV. In the meantime, the SCF tolerance of 1 × 10^−5^ eV∙atom^−1^ was adopted, with the maximum force and maximum displacement being 0.03 eV∙Å^−1^ and 0.001 Å, respectively. 

## 3. Results and Discussion

### 3.1. Synthesis and Characterization

**Figure 1:**
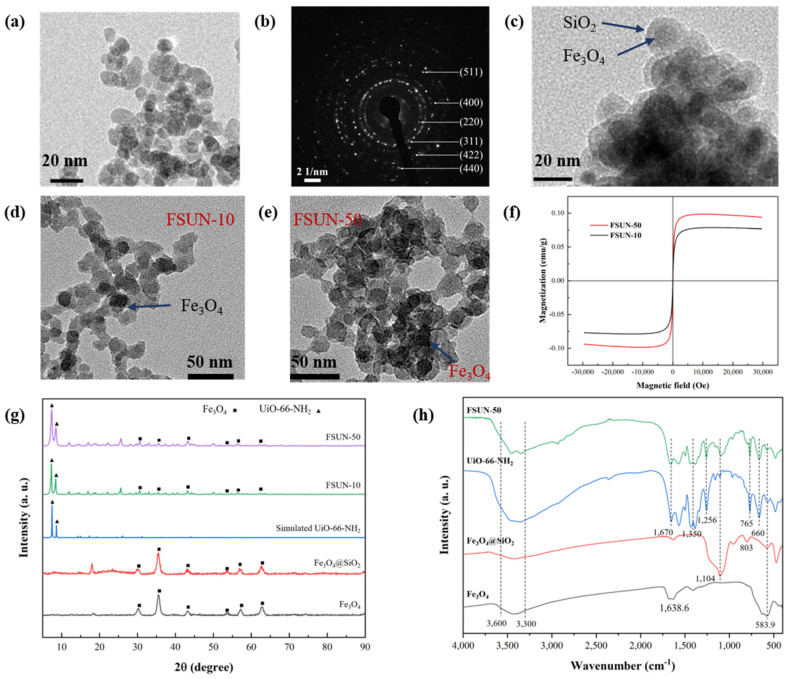
(**a**,**b**): TEM image and selected area electron diffraction of Fe_3_O_4_; (**c**–**e**): TEM images of Fe_3_O_4_@SiO_2_, FSUN–10and FSUN–50; (**f**) the magnetization curves of FSUN–10and FSUN–50; (**g**) XRD patterns of Fe_3_O_4_, Fe_3_O_4_@SiO_2_, simulated UiO–66–NH_2_, FSUN–10and FSUN–50; (**h**) IR spectra of Fe_3_O_4_, Fe_3_O_4_@SiO_2_, UiO–66–NH_2_ and FSUN–50.

The morphology of the obtained samples was characterized by TEM. The synthesized Fe_3_O_4_ nanoparticles were spherical with diameter of ~10 nm (Figure 1a). Obviously, Fe_3_O_4_ nanoparticles tended to agglomerate due to the good magnetism befitting from superparamagnetic Fe_3_O_4_. Figure 1b gives the selected area electron diffraction (SAED) of Fe_3_O_4_ nanoparticles. Fe_3_O_4_ particles presented typical polycrystalline phase characteristics, and the electron diffraction pattern was consistent well with dominant XRD diffraction peaks of crystalline Fe_3_O_4_ (Figure 1g). The TEM image of Fe_3_O_4_@SiO_2_ (Figure 1c) displayed a core-shell structure, and the silica shell could protect Fe_3_O_4_ nanoparticles in highly acidic solution, which is also the purpose of this work. Figure 1d,e show the TEM images of FSUN–10 and FSUN–50, respectively. As can be seen, Fe_3_O_4_@SiO_2_ distributed evenly in FSUN–10 while agglomerated in FSUN–50, suggesting that the amounts of magnetic nanoparticles could influence the structure of MOF composites. In the meantime, UiO–66–NH_2_ grew on the edge of magnetic nanoparticles or nanoclusters evidenced by TEM images. The EDX mapping of FSUN–50 (Figure S2) indicated that the magnetic MOF composites were composed of C, N, O, Zr, Fe and Si, which was in good agreement with the composition of chemicals used for material preparation. The magnetization curve (Figure 1f) indicated that the saturation magnetization value of FSUN–10 and FSUN–50 were measured to be 0.077 and 0.094 emu∙g^−1^, suggesting that the magnetism of MOF composites increased with the dosage of magnetic Fe_3_O_4_@SiO_2_ nanoparticles. As shown in Figure S2, a fast solid–liquid separation of FSUN can be achieved via magnetic separation (Figure S2), suggesting that the magnetic functionalized MOF composites can be retrieved in a facile manner instead of tedious high-speed centrifugation, which is beneficial to the industrial application of MOFs with lower cost and higher efficiency. 

**Figure 2:**
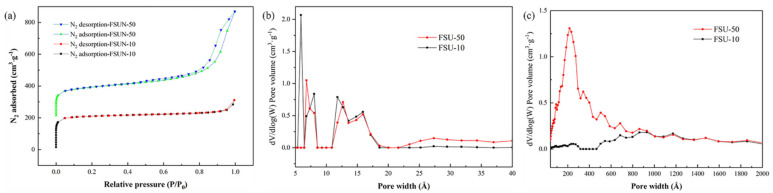
(**a**) N_2_ adsorption and desorption curves of FSUN–10 and FSUN–50; (**b**) pore size distribution of FSUN–10 and FSUN–50in the range of 5~40 Å; (**c**) pore size distribution of FSUN–10 and FSUN–50in the range of 75~2000 Å.

Figure 1g gives the XRD patterns of the obtained products. For Fe_3_O_4_, peaks at 2θ of 30.2°, 35.5°, 43.2°, 53.5°, 57.2° and 63.2° were ascribed to (2 2 0), (3 1 1), (4 0 0), (4 2 2), (5 1 1) and (4 4 0) planes of the spinal phase of Fe_3_O_4_ (JCPDS #19-0629) [23,24], indicating that the synthesized Fe_3_O_4_ possessed good crystallinity, which was consistent with the SAED results (Figure 1b). Moreover, the abovementioned characteristic peaks were also found in the XRD patterns of Fe_3_O_4_@SiO_2_, FSUN–10and FSUN–50, suggesting that Fe_3_O_4_ nanoparticles were successfully functionalized in the obtained products. The hump peaks at 2θ of 23° in the patterns of Fe_3_O_4_@SiO_2_ represented the amorphous SiO_2_ (JCPDS #39-1425) [25]. The XRD pattern of UiO–66–NH_2_ derived from crystallographic information was also presented in Figure 2. Peaks at 2θ = 7.36°, 8.48° were associated to the indices of (1 1 1) and (0 0 2) planes of the spinal phase of UiO–66–NH_2_ [21], which also existed in the patterns of FSUN–10 and FSUN–50, indicating that Fe_3_O_4_@SiO_2_ core was successfully introduced into UiO–66–NH_2_ without destroying the MOF structure.

Figure 1h shows the IR spectra of Fe_3_O_4_, Fe_3_O_4_@SiO_2_, UiO–66–NH_2_ and FSUN–50. The wide peak in the region of 3300~3600 cm^−1^ were assigned to the stretching vibrations of O-H and N-H from hydroxyl and amino groups. For the spectrum of Fe_3_O_4_, peak at 583.9 cm^−1^ was ascribed to the stretching vibration of Fe-O bond in bulk Fe_3_O_4_ [26], which could also be found in the spectrum of Fe_3_O_4_@SiO_2_ and FSUN–50. Peaks at 1104 cm^−1^ and 803 cm^−1^ were attributed to the stretching and bending vibration of Si-O-Si of SiO_2_ [27], indicating that SiO_2_ was successfully prepared in this work. As for UiO–66–NH_2_, peak at 1256 cm^−1^ referred to the stretching vibration of C-N bond, corresponding to the amino groups of NH_2_-BDC. Peaks at 765 and 660 cm^−1^ were attributed to the stretching vibration of Zr-O [28]. Furthermore, the abovementioned characteristic peaks such as Fe-O, Si-O-Si, C-N, Zr-O and benzene ring also existed in the spectrum of FSUN–50, suggesting that MOF composites composed of Fe_3_O_4_, SiO_2_ and UiO–66–NH_2_ were successfully prepared in this work, which was consistent with TEM and XRD analysis results.

The pore structure of FSUN–10 and FSUN–50was studied through N_2_ adsorption/desorption test and results are shown Figure 2. As revealed by Figure 2a, the adsorption of N_2_ onto FSUN–10 and FSUN–50complied with type I adsorption. The adsorption and desorption curves of FSUN–10 were in good agreement except at high relative pressure (P/P_0_ > 9.1) where small hysteresis loop was observed. While for the adsorption of N_2_ onto FSUN–50, big hysteresis loop existed when P/P_0_ > 0.8, suggesting that capillary condensation occurred due to the introduction of mesopores by magnetic nanoparticles. The Brunauer-Emmett-Teller (BET) surface area of FSUN–10 and FSUN–50 was 789.25 m^2^∙g^−1^ and 676.45 m^2^∙g^−^^1^, respectively, indicating that the specific surface area of magnetic functionalized MOF composites decreased with the increase in magnetic nanoparticle dosage. 

The pore volume of FSUN–10 (0.3832 cm^3^∙g^−1^) was smaller than that of FSUN–50 (0.8215 cm^3^∙g^−1^) due to the existence of mesopores in FSUN–50, verified by the subsequent pore size distribution (Figure 2b,c). The average pore diameter of FSUN–10 was 7.8 Å and 14.7 Å, corresponding to the octahedral pore (primitive cell) and cubo-octahedral pore of UiO–66–NH_2_ [29]. As for FSUN–50, in addition to the abovementioned micropores, mesopores with diameter of ~255 Å also existed, suggesting that capillary condensation occurred in FSUN–50, corresponding to the N_2_ adsorption/desorption curves (Figure 2a) and TEM images (Figure 1e).

### 3.2. Au (III) Adsorption by Magnetic Functionalized MOFs

#### 3.2.1. Effect of pH on Au (III) Adsorption

To study the effect of pH on Au (III) adsorption by FSUN–10 and FSUN–50, the initial Au (III) concentration was fixed at 400 mg∙L^−1^ and pH ranged from 1 to 9. As reported by Mironov et al. [30], Au (III) is prone to hydrolyze in aqueous solution as Equations (9) and (10):

(9) $$AuCl_{4}^{-}+H_{2}O⇌AuCl_{3}{\left(H_{2}O\right)}^{0}+Cl^{-}$$

(10) $$AuCl_{3}{\left(H_{2}O\right)}^{0}⇌AuCl_{3}OH^{-1}+H^{+}$$

Additionally, the hydrolysis equilibrium constants *K*_1_, *K*_2_ are 10^−5.1^ and 10^−0.7^, respectively. By combining Equations (9) and (10), we can get Equation (11):

(11) $$AuCl_{4}^{-}+H_{2}O⇌AuCl_{3}OH^{-}+H^{+}+Cl^{-}$$

The hydrolysis constant *K*_3_ = *K*_1_ × *K*_2_ = 10^−5.8^, and the species of Au (III) complex in aqueous solution can be determined using Equation (12):

(12) $$R=\frac{\left[AuCl_{3}OH^{-}]\cdot [Cl^{-}\right]}{\left[AuCl_{4}^{-}]\cdot [H_{2}O\right]}=\frac{K_{3}}{\left[H^{+}\right]}=\frac{10^{-5.8}}{\left[H^{+}\right]}$$

where [H_2_O] is assumed to be 1. The species of Au (III) complex in aqueous solution is determined by the pH value. In this work, the primary species of Au (III) complex in aqueous solution was AuCl_4_^−^ in acidic solution when pH was smaller than 5.8, while hydrolyzed to be AuCl_3_OH^−^ when pH was bigger than 5.8.

**Figure 3:**
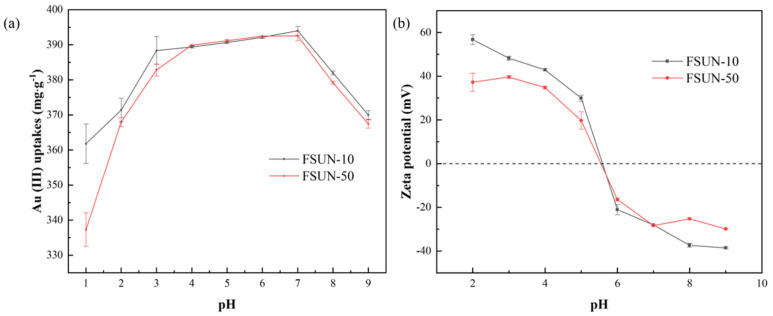
(**a**) Effect of pH on Au (III) uptakes by FSUN–10 and FSUN–50; (**b**) Effect of pH on the surface charge of FSUN–10 and FSUN–50.

Figure 3a shows that Au (III) uptakes by FSUN–10 and FSUN–50 initially increased rapidly as pH increased from 1 to 4, and the optimal adsorption was achieved at pH 4–7. After that, Au (III) uptakes decreased gradually at higher pH. It was reported by Chassary [31] that the ability of amino groups to react with Au was reduced in acidic solution due to protonation, and ion exchange and electrostatic interaction contributed to Au (III) uptakes. In this work, the interaction between AuCl_4_^−^ and -NH_3_^+^ in acidic solution are as Equation (13) [32]:

(13) $$R-NH_{3}^{+}Cl^{-}+AuCl_{4}^{-}⇌R-NH_{3}^{+}AuCl_{4}^{-}+Cl^{-}$$

As shown in Figure 3b, the pH_pzc_ of FSUN–10 and FSUN–50 was determined to be 5.58 and 5.54, respectively. Therefore, it can be concluded that in acidic solution, AuCl_4_^−^ was adsorbed by FSUN through ion exchange and electrostatic attraction, while the protonation of amino groups reduced their ability to chelate Au (III). With an increase in pH, -NH_3_^+^ gradually deprotonated and the ability of amnio groups to chelate Au (III) was recovered, thus Au (III) uptakes also increased gradually. While in alkaline solution, Au (III) anions hydrolyzed to be AuCl_3_OH^−^ and both FSUN–10 and FSUN–50 were negatively charged, the electrostatic repulsion between AuCl_3_OH^−^ and FSUN prevented Au (III) adsorption, leading to the decrease in Au (III) uptakes. Therefore, pH study indicated that the electrostatic interaction between FSUN and Au (III) played a significant role in Au (III) adsorption and acidic environment is more favorable compared to alkaline environment. 

#### 3.2.2. Adsorption Kinetics Study

**Figure 4:**
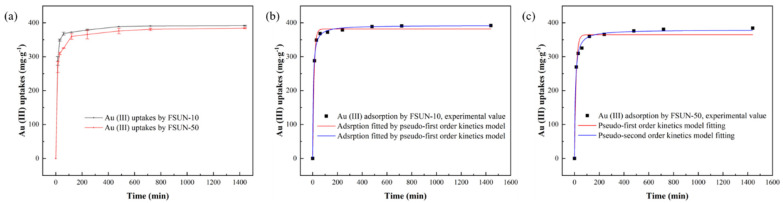
(**a**) Effect of incubation time on Au (III) uptakes by FSUN–10 and FSUN–50; (**b**) Au (III) adsorption on FSUN–10 fitted by pseudo-first order and pseudo-second order kinetics model; (**c**) Au (III) adsorption on FSUN–50 fitted by pseudo-first order and pseudo-second order kinetics model.

The adsorption kinetics of Au (III) on FSUN was studied by examining the relationship between Au (III) uptakes and incubation time. The initial Au (III) concentration was kept at 400 mg∙L^−1^ with incubation time ranging from 0 to 1440 min. As Figure 4a reveals, Au (III) uptakes initially increased rapidly in the first 60 min, then gradually flattened out and finally plateaued after 480 min. The rapid capture of Au (III) could be attributed to the developed pore structure and sufficient adsorption sites provided by UiO–66–NH_2_. Since the majority species of Au (III) in chlorinated acidic solution are AuCl_4_^−^ with effective size being 5.4 Å [33], which was much smaller than the pore diameter of FSUN–10 and FSUN–50, thus AuCl_4_^−^ could diffuse conveniently in their internal pore. In the meantime, UiO–66–NH_2_ could provide sufficient adsorption sites (i.e., amino groups) for Au (III) at the initial stage, contributing to the fast adsorption at initial stage. And the adsorption rate gradually declined as the adsorption sites were employed. 

To gain a deep insight into the adsorption kinetics, Au (III) uptakes were fitted to the incubation time using pseudo-first order kinetics model (Equation (3)) and pseudo-second order kinetics model (Equation (4)). And the adjusted fitting coefficients R^2^ and residual sum of squares (RSS) were used to evaluate the fitting reliability. Higher R^2^ and smaller RSS usually means higher predicting accuracy. Clearly, the predicted values by pseudo-second order kinetics model were closer to the experimental values than pseudo-first order kinetics model (Figure 4b,c), and R^2^ for pseudo-first order kinetics model and pseudo-second order kinetics model were 0.9951, 0.9975 for FSUN–10 and 0.9729, 0.9962 for FSUN–50 (Table 1), respectively. Moreover, the RSS values obtained by pseudo-second order kinetics model were smaller than pseudo-first order kinetics model. Therefore, pseudo-second order kinetics model could better describe the adsorption process, indicating that chemisorption was the rate-determining step.

Moreover, the kinetics constant for Au (III) uptakes by FSUN–10 were bigger than FSUN–50, suggesting that Au (III) uptakes by FSUN–10 was more quickly, verified by the shorter time taken to reach adsorption equilibrium, suggesting that the adsorption rate of Au (III) on FSUN decreased with an increase in the dosage of magnetic nanoparticles due to the reduced surface area and adsorption sites. In this work, the decrease in adsorption rate caused by magnetic functionalization was not so big and still within the acceptable range.

#### 3.2.3. Adsorption Thermodynamics Study

**Figure 5:**
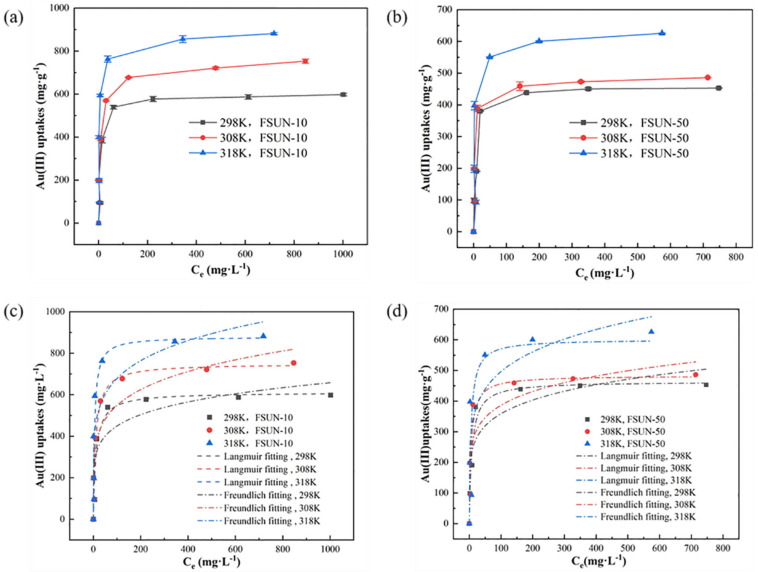
Effect of temperature on Au (III) uptakes by FSUN–10 (**a**) and FSUN–50 (**b**); Au (III) adsorption on FSUN–10 (**c**) and FSUN–50 (**d**) fitted by Langmuir model and Freundlich model.

To study the effect of initial concentration and temperature on Au (III) uptakes by FSUN, the adsorption test was conducted with initial Au (III) concentration varying from 0 to 1600 mg∙L^−1^, temperature ranging from 298 K to 318 K, and the incubation time was fixed at 1440 min. As shown in Figure 5a,b, Au (III) uptakes initially increased significantly as Au (III) concentration increased from 0 to 600 mg∙L^−1^, then the increase gradually slowed down and finally plateaued when reaching 800 mg∙L^−1^. In the meantime, Au (III) uptakes by FSUN increased with temperature, suggesting that the adsorption process was endothermic. Moreover, Au (III) uptakes by FSUN–10 and FSUN–50 were close at low initial concentration, while the difference in Au (III) uptakes enlarged with increasing initial concentration. This is because FSUN–10 could provide more adsorption sites for Au (III) than FSUN–50 due to less amounts of magnetic nanoparticles were introduced. Furthermore, Au (III) uptakes at different temperature were fitted to the residual Au (III) concentration using Langmuir and Freundlich adsorption models, and the fitting reliability was also evaluated by R^2^ and RSS. For both FSUN–10 and FSUN–50, Langmuir model fitted better than Freundlich model (Figure 5c,d), verified by bigger R^2^ and smaller RSS values obtained through Langmuir fitting (Table 2). Thus, it confirms that the adsorption isotherms of Au (III) by FSUN–10 and FSUN–50 were in better agreement with the Langmuir adsorption model, suggesting that Au (III) uptakes by FUSN were monolayer and homogeneous. 

The maximum uptakes of Au (III) on some nanomaterials reported in the literature are summarized in Table 3. Obviously, compared to the pristine UiO–66–NH_2_ synthesized by authors in the previous work, the adsorption abilities of Zr-MOF composites decreased slightly after magnetic functionalization, which is expectable since magnetic functionalization reduced the overall surface area and the adsorption ability of Fe_3_O_4_@SiO_2_ towards Au (III) was smaller than MOFs [34]. This work is dedicated to achieving a convenient retrieval of MOF composites by sacrificing adsorption capacity within acceptable range. In the meantime, the magnetic functionalized MOF composites displayed superiority compared to other nanomaterials, which could be attributed to the developed pore structure, large surface area and abundant functional groups, thereby endowing magnetic MOFs sufficient adsorption sites and high binding ability for Au (III). Moreover, the convenient retrieval of magnetic functionalized MOF composites makes them promising in industrial application.

### 3.3. Mechanism Study

As presented above, FUSN showed excellent adsorption ability towards Au (III). Magnetic properties after Fe_3_O_4_@SiO_2_ functionalization rendered the composites enhanced operability indicated by easy separation. The overwhelmingly advantageous adsorption towards Au (III) can be ascribed to the inherent accessible pores in UiO–66–NH_2_. As FSUN–50 has balanced magnetic properties and good adsorption ability, it was selected as a more promising candidate for industrial application. Therefore, we used FSUN–50 as the model to investigate the underlying adsorption mechanism.

**Figure 6:**
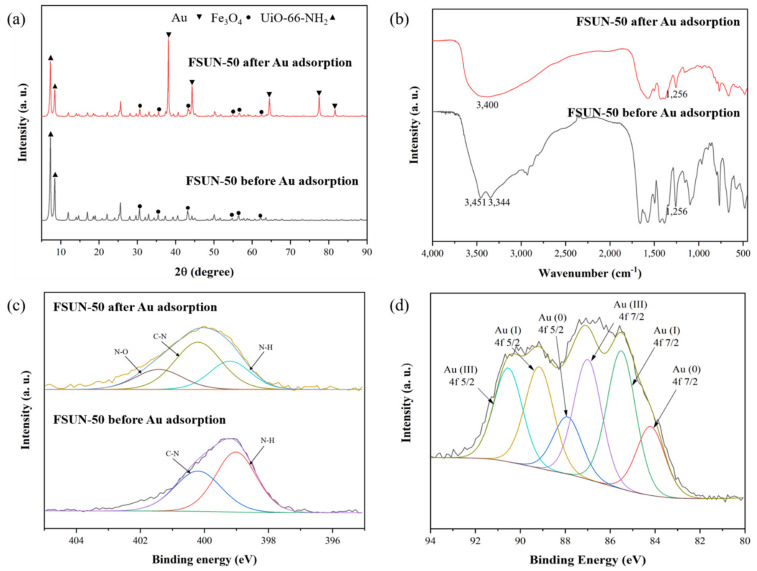
(**a**) XRD patterns and (**b**) IR spectra of FSUN–50 before and after Au (III) adsorption; high resolution scan of (**c**) N 1s and (**d**) Au 4f of FSUN–50 before and after Au (III) adsorption.

XRD patterns in Figure 6a show that new peaks were generated at 2θ = 38.18°, 44.39°, 64.56°, 77.55° and 81.72° after Au (III) adsorption, which were assigned to the (1 1 1), (0 0 2), (0 2 2), (1 1 3) and (2 2 2) planes of Au (0) (JCPDS #04-0784), respectively [16], indicating that Au (III) was reduced to Au (0) during the adsorption process. By comparing the IR spectra of FSUN–50 before and after Au (III) adsorption (Figure 6b), it was found that the stretching vibration of -OH and -NH at 3451 cm^−1^ and 3344 cm^−1^ merged into one wide peak at 3400 cm^−1^, which could be associated to the coordination of Au (III) to hydroxyl and amino groups [29]. Moreover, the stretching vibration of C-N at 1256 cm^−1^ decreased sharply after Au (III) adsorption, implying that amino groups was responsible for the reduction of Au (III) during adsorption due to its electron-donating property.

To further investigate the chemical interaction between Au (III) and amino groups, XPS survey scan and high-resolution scan of Au 4f and N 1s for FSUN–50 before and after Au (III) adsorption was conducted. XPS survey scan (Figure S3) results indicated that, before Au (III) adsorption, the synthesized FSUN–50 primarily consisted of elements including O, N, C, Zr and Si. Because XPS could only detect top 20 atomic layers which is roughly 10 nm, Fe located at the inner core was not found in the XPS survey scan spectra. In the meantime, after Au (III) adsorption, new peaks representing Cl 2p and Au 4f were detected by XPS survey scan, which could be ascribed to the adsorption of Au (III) chloride complex ions. In the high-resolution scan of N 1s (Figure 6c), the N 1s peak before Au (III) adsorption could be deconvoluted into peaks including N-H and C-N at 399.0 eV and 400.2 eV, respectively [39]. After Au (III) adsorption, peak representing N-H reduced significantly while a new peak emerged at 401.5 eV, which was attributed to the N-O bond. The results suggested that amino groups were oxidized during Au (III) adsorption, corresponding to the IR spectrum. Moreover, the high-resolution scan of Au 4f for FSUN–50 (Figure 6d) after adsorption showed that Au 4f peak could be further deconvoluted into peaks at 90.6 eV, 89.2 eV, 88.0 eV, 87.0 eV, 85.5 eV and 84.2 eV, corresponding to Au (III) 4f 5/2, Au (I) 4f 5/2, Au (0) 4f 5/2, Au (III) 4f 7/2, Au (I) 4f 7/2 and Au (0) 4f 7/2, respectively [40,41]. The results indicated that part of the adsorbed Au (III) was reduced to Au (I) and Au (0). By calculating the surface area of each sub-peak, the ratio of Au (0), Au (I) and Au (III) occupied 18.02%, 34.26% and 47.72, respectively. Thus, about 52.28% of Au (III) was reduced during adsorption. 

**Figure 7:**
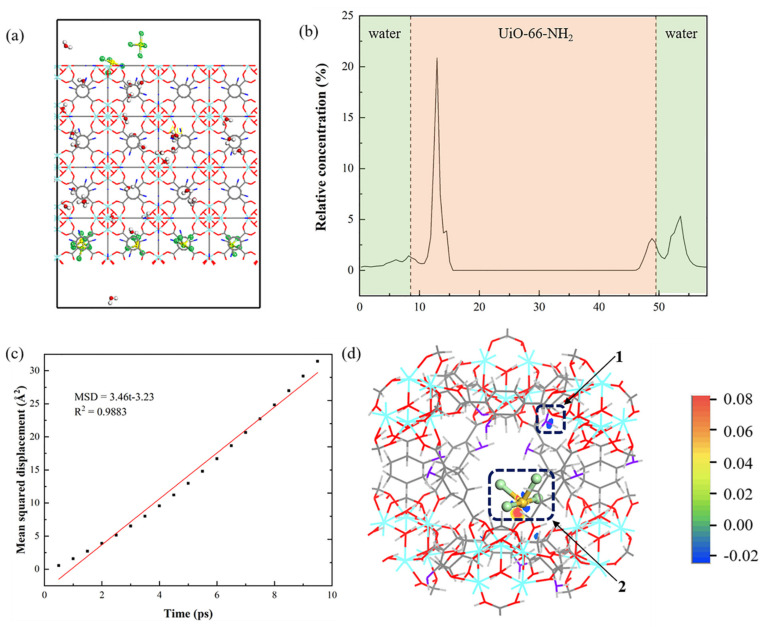
(**a**) The configuration after Au (III) adsorption with lowest energy; (**b**) the concentration profile of Au along the (0 0 1) plane of UiO–66–NH_2_; (**c**) the mean squared displacement and the fitted line of Au on UiO–66–NH_2_; (**d**) electron density difference of bonding motif after AuCl_4_^−^ adsorption on UiO–66–NH_2_; the atoms of hydrogen, oxygen, nitrogen, carbon, zirconium, gold and chloride are colored as white, red, blue, grey, cyan, yellow and green, respectively.

With all these finding based on experimental data analysis, MDS and DFT study were performed to further study the adsorption behavior of AuCl_4_^−^ on UiO–66–NH_2_ at the atomic and molecular level. Figure 7a shows the final configuration of the adsorption system with lowest energy. AuCl_4_^−^, part of water molecules and H^+^ entered the UiO–66–NH_2_ framework, suggesting that AuCl_4_^−^ could diffuse along the internal pore of UiO–66–NH_2_ and thus being adsorbed. Figure 7b gives the concentration profile of Au along the z axis of UiO–66–NH_2_ (0 0 1) plane, which could be further divided into three area, including the aqueous solution on both sides (green area, 0–8.5 Å and 49.5–58 Å) and UiO–66–NH_2_ in the middle (orange area, 8.5–49.5 Å). Peaks at z = 12.97 Å and 48.88 Å demonstrated that Au (III) entered into UiO–66–NH_2_, which agreed well with Figure 7a. To investigate the diffusion coefficient of AuCl_4_^−^ along the framework of UiO–66–NH_2_, the mean squared displacement (MSD) of Au during the simulation process was studied and results are shown in Figure 7c. The MSD data were fitted by the linear model. According to Equation (8), the diffusion coefficient of Au (III) was determined to be 5.8 × 10^−5^ cm^2^∙s^−^^1^.

Figure 7d shows the electron density difference of bonding atoms after AuCl_4_^−^ adsorption on UiO–66–NH_2_. The red and blue color means electron acceptance and loss, respectively. N atoms from amino groups lost electrons during adsorption (area 1) while Au atoms accepted electrons (area 2), confirming that electrons transferred from amino groups to Au (III). It was verified in theory that amino groups contributed to the reduction of Au (III) to Au (I) and Au (0) during the adsorption process, which is in good correspondence with IR, XRD and XPS results.

Based on the previous findings achieved by batch adsorption test, experimental characterization and computational modelling, mechanisms underpinning Au (III) adsorption on FSUN could be summarized as follows. Initially, FSUN provides accessible pores and allows Au (III) to diffuse along its framework due to the developed pore structure and appropriate pore diameter. Then, Au (III) is adsorbed through ion exchange and is coordinated to the hydroxyl and amino groups, facilitated by the electrostatic attraction between -NH_3_^+^ and Au (III) anions. For Au (III) coordinated to amino groups, electrons transfer from amino groups to Au (III), resulting in the in-situ reduction of Au (III) to Au (I) and Au (0). Overall, the mechanism of Au (III) adsorption by FSUN incorporates diffusion, ion exchange, coordination, electrostatic interaction and reduction, and chemisorption is the rate-determining step, evidenced by the adsorption kinetics study.

### 3.4. Selectivity Study

Since Au (III) always coexist with impurity metal ions such as Cu^2+^, Ni^2+^, Co^2+^ and Zn^2+^ in aqueous solution after extraction of gold from e-waste through hydrometallurgy, the selectivity towards Au (III) is an important factor that must be considered in practice. In this work, to study the selectivity of magnetic MOFs, AuCl_4_^−^ was mixed with Cu^2+^, Ni^2+^, Co^2+^ and Zn^2+^, and the concentration of all the metal ions was fixed at 200 mg∙L^−1^. Then, FSUN was incubated with the mixed solution to test the adsorption selectivity. The uptakes of Au (III) by FSUN–10 and FSUN–50 were 199.22 mg∙g^−1^ and 188.09 mg∙g^−1^, while the uptakes of Co^2+^, Cu^2+^, Ni^2+^ and Zn^2+^ by FSUN were all smaller than 50 mg∙g^−1^ (Figure S5), suggesting that FSUN possessed excellent adsorption priority towards Au (III) rather than impurity metal ions, which could be attributed to two reasons. At first, as shown in Figure 3b, FSUN were positively charged in the pH range of 1–5.6, preferring to adsorb AuCl_4_^−^ due to electrostatic attraction, while the electrostatic repulsion between FSUN and cationic metal ions Cu^2+^, Ni^2+^, Co^2+^ and Zn^2+^ prevented the adsorption of co-existing metal ions. Secondly, as mentioned above, Au (III) was reduced to Au (I) and Au (0) by amino groups during adsorption, and the standard electrode potential of Au (III)|Au (I) and Au (III)|Au (0) was 1.50 eV and 1.41 eV, respectively, suggesting that Au (III) was prone to be reduced to Au (I) and Au (0). However, the standard electrode potential of Cu^2+^|Cu (0), Ni^2+^|Ni (0), Co^2+^|Co (0), Zn^2+^|Zn (0) was 0.34 eV, −0.25 eV, −0.28 eV, and −0.7628 eV, which was much smaller than Au (III)|Au (I) and Au (III)|Au (0). Therefore, the reduction of co-existing ions during adsorption was almost impossible. FSUN possessed high priority towards Au (III) than impurity metal ions in the adsorption process, making them ideal candidates as Au (III) adsorbent in practice.

### 3.5. Reusability Study

Reusability is a key factor that must be considered when assessing the availability of adsorbents in practice. In this work, the reusability of FSUN–10 and FSUN–50 was evaluated by recycling tests. As shown in Figure S6 for FSUN–10, the Au (III) uptakes decreased from 33.03 mg∙g^−1^ to 23.98 mg∙g^−1^ after five cycles, and the regenerated adsorbents retained 72.60% of the initial adsorption capacity. FSUN–50 performed better than FSUN–10 and 76.06% of initial adsorption ability remained after recycling five times. In addition, the TEM image of regenerated FSUN–50 (Figure S7) showed that no big changes happened to the micromorphology of this adsorbent, suggesting that FSUN had high stability during Au (III) adsorption. Overall, the reusability results indicated that FSUN displayed good adsorption ability towards Au (III) after multiple regeneration, commodifying the feasibility and excellent reusability of MOF composites in practice.

## 4. Conclusions

In this work, magnetic MOF composites were prepared to effectively adsorb Au (III) from aqueous solution and favor adsorbents retrieval. Effects of magnetic functionalization on the structural properties and adsorption performance of MOF composites, as well as the adsorption mechanisms were systematically investigated. Overall, magnetic functionalization introduced mesopores into MOF composites and enabled them to be rapidly retrieved via magnetic separation. pH was critical to Au (III) adsorption on FSUN by governing the ion exchange and electrostatic interaction between Au (III) anions and adsorbents, and the optimal adsorption capacities were achieved at pH 7. The kinetics and isotherms of Au (III) adsorption could be described by pseudo-second order kinetics model and Langmuir adsorption model, respectively. The maximum adsorption capacities of Au (III) by FSUN–10 and FSUN–50 were 611.18 mg∙g^−1^ and 463.85 mg∙g^−1^, respectively, which were slightly smaller than the pristine UiO–66–NH_2_ due to the reduced surface area and smaller adsorption capacity of Fe_3_O_4_@SiO_2_. Additionally, Au (III) uptakes increased with temperature, indicating that Au (III) adsorption on FSUN was endothermal. The thermodynamic studies indicated that the adsorption of Au (III) on FSUN was driven by the increase in system entropy. The adsorption mechanisms were investigated systematically through material characterization, MDS and DFT study. In addition to ion exchange and electrostatic interaction, Au (III) could be adsorbed via coordination to hydroxyl and amino groups and reduced to Au (I) and Au (0) by amino groups, which was verified by IR spectrum, XPS analysis and DFT study. The diffusion coefficient of AuCl_4_^−^ in UiO–66–NH_2_ was calculated to be 5.8 × 10^−5^ cm^2^∙s^−1^. Moreover, FSUN showed excellent selectivity towards Au (III) and high adsorption ability after five cycles. FSUN–50, evaluated with balanced magnetic properties and adsorption capability, was considered to be a promising candidate applicable in industrial Au (III) recovery.

## Figures and Tables

**Table 1:** Kinetics parameters for Au (III) adsorption by FSUN–10 and FSUN–50.

Kinetics Model	Parameters	FSUN–10	FSUN–50
Pseudo-first order kinetics model	q_e_/(mg·g^−1^)	381.55	364.79
	k_1_/(min^−1^)	0.09	0.0787
	R^2^	0.9951	0.9729
	RSS	546.31	2806.34
Pseudo-second order kinetics model	q_e_/(mg·g^−1^)	392.65	379.51
	k_2_/(g·mg^−1^·min^−1^)	5.18 × 10^−4^	3.94 × 10^−4^
	R^2^	0.9975	0.9962
	RSS	282.55	391.17

**Table 2:** Isotherm parameters for Au (III) uptakes by FSUN–10 and FSUN–50.

T/K	Adsorption Model	Parameters	FSUN–10	FSUN–50
**298 K**	**Langmuir**	K_L_/(L·mg^−1^)	0.0853	0.1213
		q_m_/(mg·g^−1^)	611.18	463.85
		R^2^	0.8813	0.9629
		RSS	41,983.88	6580.85
	**Freundlich**	K_F_	209.52	164.43
		*n*	6.04	5.90
		R^2^	0.8232	0.8581
		RSS	625,272.83	25,204.66
**308 K**	**Langmuir**	K_L_/(L·mg^−1^)	0.0941	0.1788
		q_m_/(mg·g^−1^)	749.29	485.97
		R^2^	0.9289	0.8583
		RSS	37,860.05	28,304.90
	**Freundlich**	K_F_	230.39	185.77
		*n*	5.31	6.29
		R^2^	0.8514	0.8122
		RSS	79,124.61	37,530.88
**318 K**	**Langmuir**	K_L_/(L·mg^−1^)	0.1807	0.1834
		q_m_/(mg·g^−1^)	879.64	601.56
		R^2^	0.7941	0.7399
		RSS	151,483.98	85,143.14
	**Freundlich**	K_F_	306.98	220.26
		*n*	5.81	5.67
		R^2^	0.7516	0.6560
		RSS	182,795.22	112,598.62

**Table 3:** Maximum Au (III) uptakes by nanomaterials, T = 298 K.

Nanomaterials	q_max_ (mg∙g^−1^)	Ref.
UiO–66–NH_2_	650	[16]
FSUN–10	611.18	This work
FSUN–50	463.85	This work
UiO-66-TU	326	[35]
Activated carbon	35.88	[36]
Silica gel	459.01	[37]
Glycine modified crosslinked chitosan resin	169.98	[32]
Fe_3_O_4_@SiO_2_	128.84	[38]

## Data Availability

Not applicable.

## Supplementary Materials

The following supporting information can be downloaded at: https://www.mdpi.com/article/10.3390/ma15196531/s1, Figure S1: The initial configuration of AuCl_4_-, H_2_O, H^+^ and UiO–66–NH_2_ before adsorption dynamics simulation. The atoms of hydrogen, oxygen, nitrogen, carbon, zirconium, gold and chloride are colored as white, red, blue, grey, cyan, yellow and green; Figure S2: (a) EDX elemental mapping background of FSUN–50; (b–h): elemental distribution of C, O, Zr, Si, N of FSUN–50.; Figure S3: Magnetic separation of FSUN–10 (a) and FSUN–50 (b) by external magnet; Figure S4: XPS survey scan of FSUN–50 before and after Au (III) adsorption; Figure S5: Adsorption of Au (III), Co^2+^, Cu^2+^, Ni^2+^ and Zn^2+^ by FSUN–10 and FSUN–50; Figure S6: Au (III) uptakes by FSUN–10 and FSUN–50 as a function of recycling time (m = 30 mg, V = 10 mL, C_0_ = 100 mg∙L^−1^); Figure S7: TEM image of the regenerated FSUN–50.
