# Supplementary material for: Magnetic Zr-Based Metal-Organic Frameworks: A Highly Efficient Au (III) Trapper for Gold Recycling
Source: Materials (Basel). 2022 Sep 21;15(19):6531. doi: 10.3390/ma15196531 (PMC9572273; doi:10.3390/ma15196531)
Supplement: Supplementary file 1 [file materials-15-06531-s001.zip › materials-1901334-supplementary.pdf]

Magnetic functionalized Zr-based MOFs for effective adsorption of Au (III) from aqueous solution and mechanism study

Ziyong Chang [1,*], Xiaosha Gong [1], Liang Zeng [1], Junlian Wang [1] and Yangge Zhu [2,*]

[1] Civil and Resource Engineering School, University of Science and Technology Beijing, Beijing 100083, China
[2] BGRIMM Technology Group, State Key Laboratory of Mineral Processing, Beijing 100160, China
* Correspondence: changziyong@ustb.edu.cn (Z.C.); zhuyangge@bgrimm.com (Y.Z.); Tel.: +86-138-1021-5948 (Z.C.); Tel.: +86-187-0168-9731 (Y.Z.)

Materials and instrumentation

Zirconium tetrachloride ($ZrCl_4$) was purchased from Shanghai Meryer Chemical Technology Co. Ltd., 2-aminoterephthalic acid (NH_2-BDC), N,N-dimethyformamide (DMF) and tetrachloroauric acid trihydrate ($HAuCl_4{\cdot}3H_2O$) were supplied by Aladdin Co. Ltd., ferric chloride hexahydrate ($FeCl_3{\cdot}6H_2O$), sodium bicarbonate ($NaHCO_3$), Vitamin C and tetraethyl orthosilicate (TEOS) were provided by J&K Scientific (Beijing) Co. Ltd., glacial acetic acid (CH_3COOH) and ethanol were supplied by Tongguang Chemical Technology (Beijing) Co. Ltd.. Chemicals used in this work were all used in their as-received states without further purification.

The magnetic functionalized MOFs before and after Au (III) adsorption were characterized by Fourier Transform Infrared Spectroscopy (FTIR, Thermo Nicoiet), Malvern Zetasizer Nano ZS90, powder X-ray diffraction analyzer (PXRD, Rigaku Miniflex600, Cu Kα radiation, 40 kV, 40 mA), transmission electron microscopy (TEM, JEOL, JEM-2200FS) and X-ray photoelectron spectroscopy (XPS, Kratos, Al Kα radiation). The concentration of Au (III) after adsorption was determined using Inductively Coupled Plasma Optical Emission Spectrometer (ICP-OES, iCAP7000 plus).

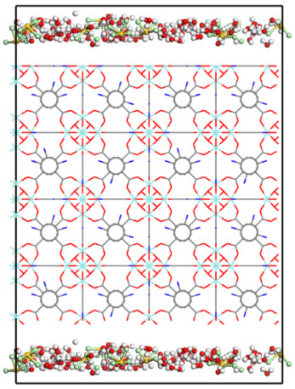

Figure S1. The initial configuration of $AuCl_4^-$, H_2O, H^+ and UiO-66-NH_2 before adsorption dynamics simulation. The atoms of hydrogen, oxygen, nitrogen, carbon, zirconium, gold and chloride are colored as white, red, blue, grey, cyan, yellow and green.

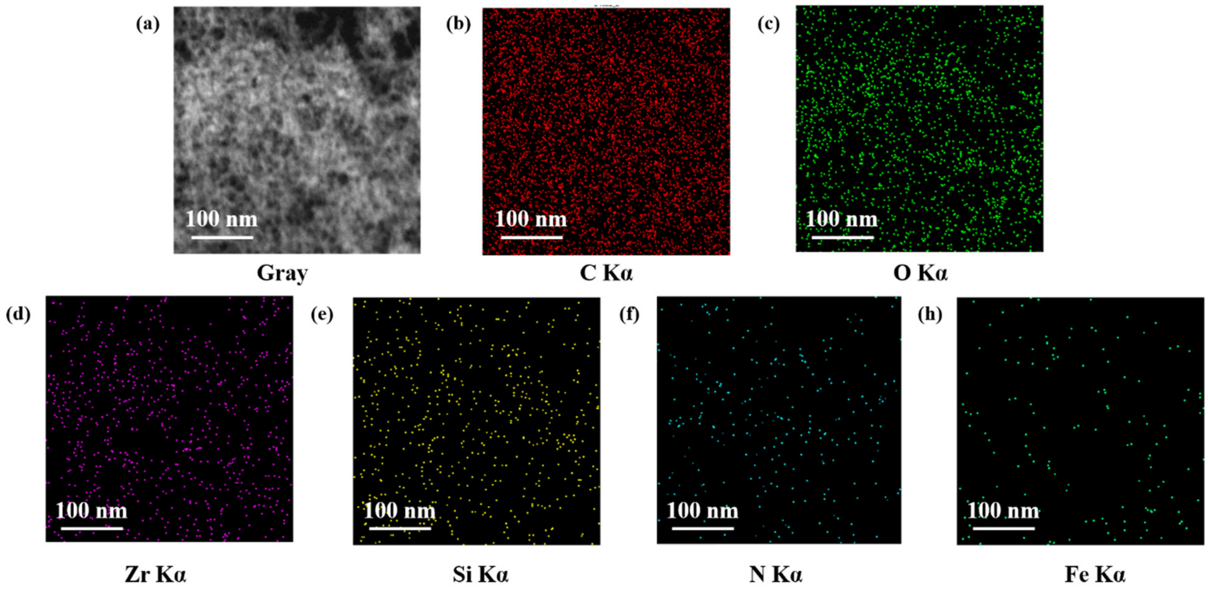

Figure S2. (a) EDX elemental mapping background of FSUN-50; (b–h): elemental distribution of C, O, Zr, Si, N of FSUN-50.

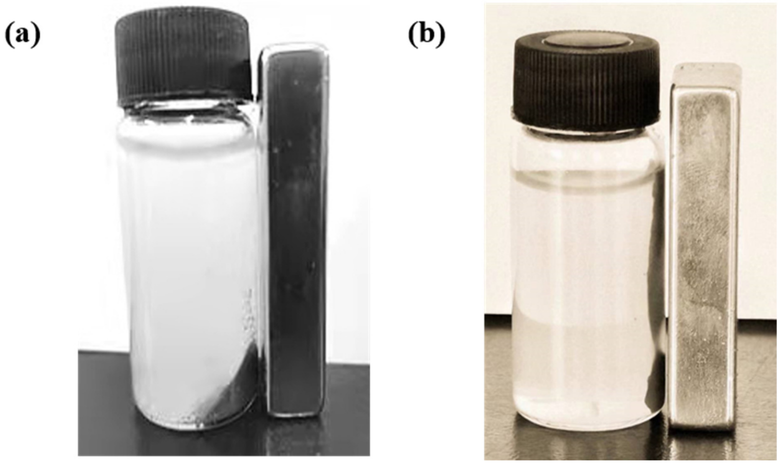

Figure S3. Magnetic separation of FSUN-10 (a) and FSUN-50 (b) by external magnet.

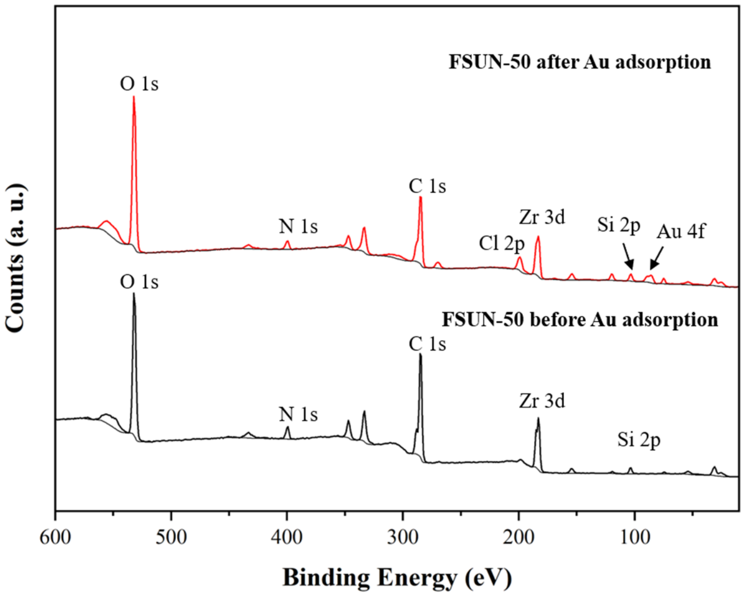

Figure S4. XPS survey scan of FSUN-50 before and after Au (III) adsorption.

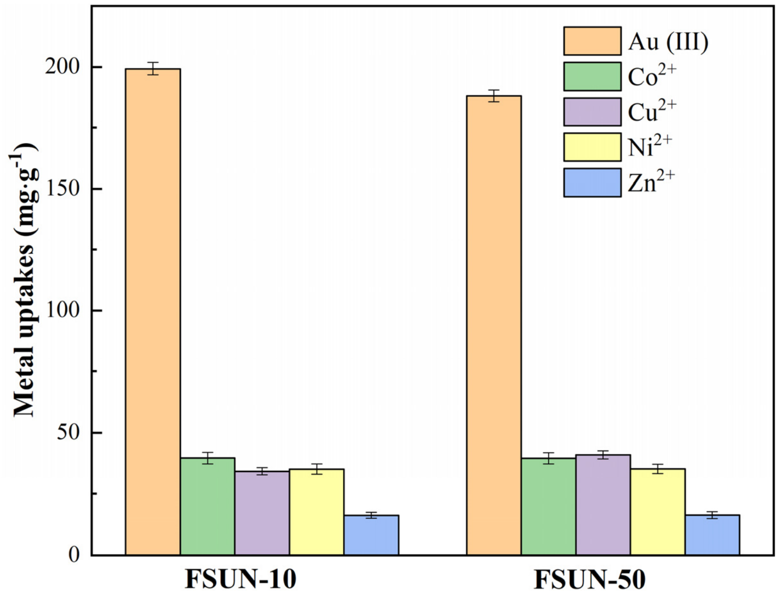

Figure S5. Adsorption of Au (III), Co^{2+}, Cu^{2+}, Ni^{2+} and Zn^{2+} by FSUN-10 and FSUN-50.

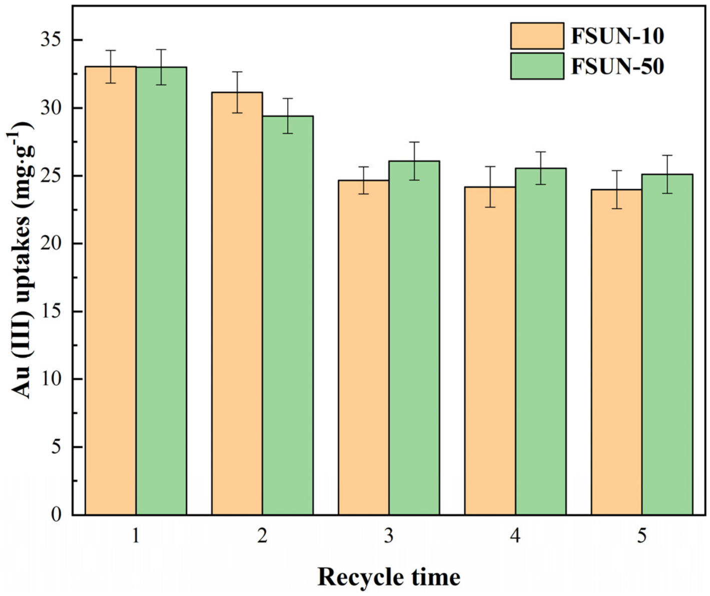

Figure S6. Au (III) uptakes by FSUN-10 and FSUN-50 as a function of recycling time (m = 30 mg, V = 10 mL, C_0 = 100 mg·L^{-1}).

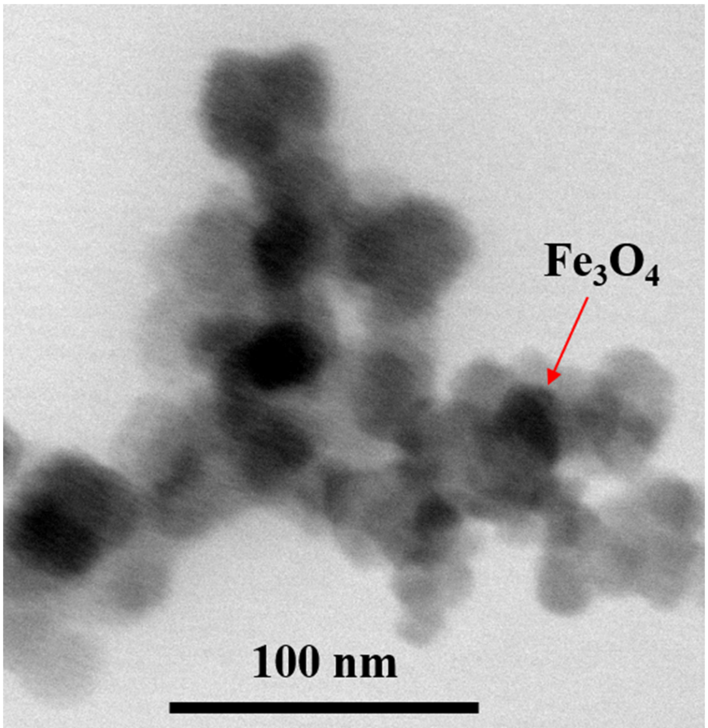

Figure S7. TEM image of the regenerated FSUN-50.
